# H4K5 Butyrylation Coexist with Acetylation during Human Spermiogenesis and Are Retained in the Mature Sperm Chromatin

**DOI:** 10.3390/ijms232012398

**Published:** 2022-10-17

**Authors:** Alberto de la Iglesia, Paula Jauregi, Meritxell Jodar, Ferran Barrachina, Lukas Ded, Carme Mallofré, Leonardo Rodríguez-Carunchio, Juan Manuel Corral, Josep Lluís Ballescà, Katerina Komrskova, Judit Castillo, Rafael Oliva

**Affiliations:** 1 Molecular Biology of Reproduction and Development Research Group, Institut d’Investigacions Biomèdiques August Pi i Sunyer (IDIBAPS), Fundació Clínic per a la Recerca Biomèdica, Departament de Biomedicina, Facultat de Medicina i Ciències de la Salut, Universitat de Barcelona (UB), 08036 Barcelona, Spain; 2 Biochemistry and Molecular Genetics Service, Clínic Barcelona, 08036 Barcelona, Spain; 3 Laboratory of Reproductive Biology, Institute of Biotechnology, Czech Academy of Sciences, BIOCEV, 252 50 Vestec, Czech Republic; 4 Department of Pathology, Clínic Barcelona, 08036 Barcelona, Spain; 5 Faculty of Medicine, University of Vic-Central University of Catalonia (UVic-UCC), 08500 Barcelona, Spain; 6 Clinic Institute of Urology and Nephrology, IDIBAPS, Clínic Barcelona, 08036 Barcelona, Spain; 7 Clinic Institute of Gynaecology, Obstetrics and Neonatology, Clínic Barcelona, 08036 Barcelona, Spain; 8 Department of Zoology, Faculty of Science, Charles University, 128 44 Prague, Czech Republic

**Keywords:** butyrylation, acetylation, H4K5, spermatogenesis, sperm, sperm chromatin, epigenetic regulation

## Abstract

Male germ cells experience a drastic chromatin remodeling through the nucleo-histone to nucleo-protamine (NH-NP) transition necessary for proper sperm functionality. Post-translational modifications (PTMs) of H4 Lys5, such as acetylation (H4K5ac), play a crucial role in epigenetic control of nucleosome disassembly facilitating protamine incorporation into paternal DNA. It has been shown that butyrylation on the same residue (H4K5bu) participates in temporal regulation of NH-NP transition in mice, delaying the bromodomain testis specific protein (BRDT)-dependent nucleosome disassembly and potentially marking retained nucleosomes. However, no information was available so far on this modification in human sperm. Here, we report a dual behavior of H4K5bu and H4K5ac in human normal spermatogenesis, suggesting a specific role of H4K5bu during spermatid elongation, coexisting with H4K5ac although with different starting points. This pattern is stable under different testicular pathologies, suggesting a highly conserved function of these modifications. Despite a drastic decrease of both PTMs in condensed spermatids, they are retained in ejaculated sperm, with 30% of non-colocalizing nucleosome clusters, which could reflect differential paternal genome retention. Whereas no apparent effect of these PTMs was observed associated with sperm quality, their presence in mature sperm could entail a potential role in the zygote.

## 1. Introduction

Histone post-translational modifications (PTMs) play an essential role in epigenetic regulation of spermiogenesis, the last phase of spermatogenesis, marked by a unique process of chromatin remodeling of male germ cell’s nucleus that is necessary for proper sperm function [1,2,3,4,5,6,7,8]. In mammals, a histone hyperacetylation wave concomitant to nucleosome disassembly is necessary for gradual protamine incorporation into paternal genome through the process of nucleo-histone to nucleo-protamine (NH-NP) transition [9,10,11,12,13,14]. Acetylation of histone tails contributes to loosen both histone-DNA and nucleosome-nucleosome interactions [12,15], allowing the incorporation of histone variants and transition proteins, that also contribute to chromatin relaxation for protamine recruitment and processing [13,16,17,18,19,20,21]. The residues Lys5 and Lys8 of histone H4 (H4K5 and H4K8) are of special importance in the regulation of NH-NP transition since acetylation on these amino acids (H4K5ac and H4K8ac, respectively) is recognized by the first bromodomain of the bromodomain-containing testis specific protein BRDT, which entails the first step for nucleosome instability and genome-wide histone eviction [22,23,24,25,26,27,28].

Acetylation of H4 has been widely studied in normal and pathogenic testes within the context of NH-NP transition and has been recently found altered in the testis of patients affected by testicular tumors [29,30,31,32]. Of note, the presence of additional H4 PTMs also belonging to the group of histone acylations, with similar physicochemical properties as acetylation, have also been identified in the testis. However, their potential role during human spermatogenesis is not well characterized yet [33,34,35]. Among them, histone butyrylation arises as an interesting epigenetic regulator of NH-NP transition. Contrary to what could be expected from the general role of histone acylations stimulating transcription and, therefore, inducing open chromatin states, BRDT seems to be blind to butyrylated H4K5 (H4K5bu), making H4K5bu-bearing nucleosomes able to escape histone eviction at the time of the hyperacetylation wave in mice [36]. Moreover, this behavior takes place regardless of the presence of butyrylation or acetylation on H4K8, which points out H4K5 as the main specific residue of this mechanism. Therefore, it has been suggested a temporal regulation of delayed histone replacement of nucleosomes marked by H4K5bu, which could also underlie a specific mechanism of nucleosome retention in mature sperm [36]. During mouse spermatogenesis, H4K5 acetylation and butyrylation also coexist in different spermatogenic stages, being enriched at regions surrounding transcription start sites of active genes in a transcription-dependent manner [36].

The importance of histone PTMs in male germ cells is not limited to their regulatory role during chromatin remodeling. Sperm cells contain many layers of epigenetic information, with modified histones contributing to the non-random distribution of the nucleo-histone genomic domain along specific regions of the paternal genome and marking differential sperm DNA accessibility [2,5,37,38,39,40]. In addition, infertile men evidence alterations of histone PTM profile and impaired nucleosome retention in mature sperm [2,4,41]. Sperm epigenetic marks are susceptible to be transmitted to the zygote during fecundation, potentially contributing to early stages of embryo development and future offspring health [42,43,44,45].

One of the current challenges of assisted reproduction techniques (ART) relies on selecting the best spermatozoa for intracytoplasmic sperm injection (ICSI), overcoming the intrinsic high heterogeneity of the human sperm sample. Current methods for sperm selection are based on multilayer density gradient centrifugation, often followed by swim-up. However, these techniques mainly rely on the quality of sperm motility and morphology not accounting on the rest of sperm molecular features that could affect the ART success rate (around 30–35% per cycle in Europe) [46]. In terms of sperm chromatin, sperm populations selected through density gradient centrifugation seem to show different retention of histone PTMs and histone variants compared to the corresponding neat sample [47]. However, the effect of density gradient centrifugation on other sperm quality parameters, such as DNA integrity as a direct indicator of sperm chromatin maturity, is unclear, with some articles reporting subgroups of patients with increased DNA damage after sperm preparation [48,49,50]. Therefore, there is unmet need for markers of sperm quality.

Despite the relevance of the H4K5 residue in the regulation of mouse NH-NP transition, there is a lack of information about the significance and distribution of acetylated and butyrylated forms in normal human spermatogenesis and testicular pathologies, as well as about their possible retention in the mature human spermatozoon. Therefore, the aim of our work was to increase the current knowledge about these specific-residue PTMs in human male germ cells, as well as to try to decipher whether they could be markers of human sperm quality. We report a different behavior of H4K5bu and H4K5ac during human spermatogenesis, which would indicate a highly specific role of H4K5bu during round spermatid (rSPD) elongation, coexisting with H4K5ac during spermiogenesis. Results in patients with testicular cancer evidence that H4K5ac and H4K5bu remain stable under pathological conditions, supporting the important role of these residue-specific modifications towards spermatogenic development. Of note, both H4K5bu and H4K5ac are retained in ejaculated sperm showing differential location in 30% of nucleosome clusters. Their role as sperm chromatin maturity markers after sperm selection through density gradient centrifugation has also been explored.

## 2. Results

### 2.1. H4K5 Butyrylation Show Different Spermatogenic Pattern to Acetylation on the Same Residue in Patients with Normal Spermatogenesis

**Figure 1:**
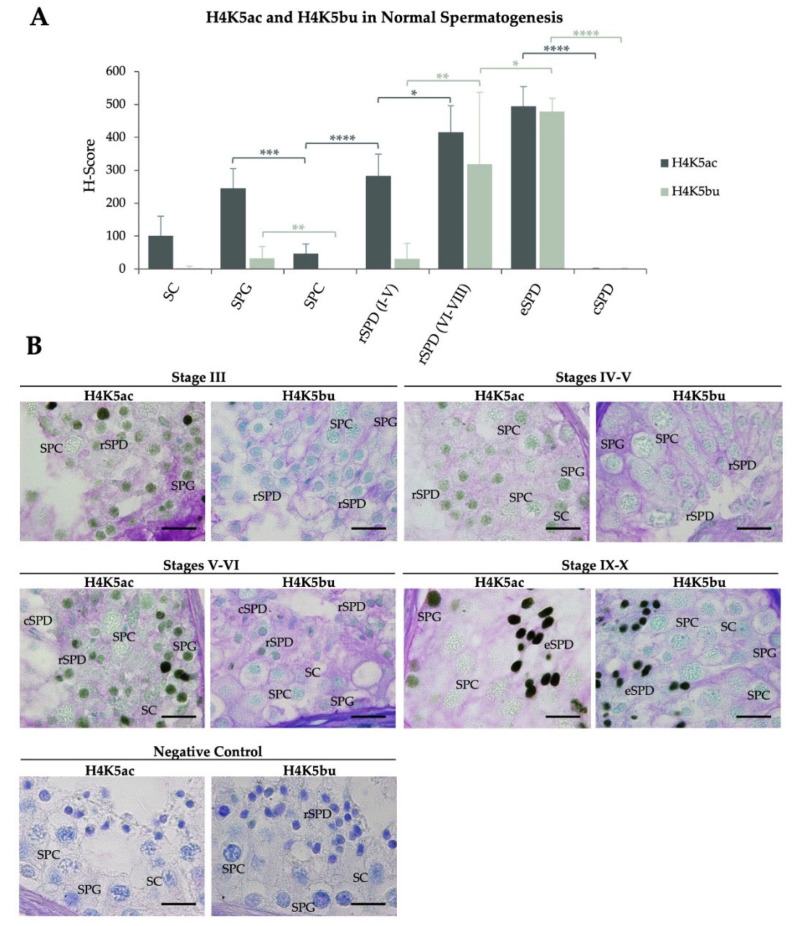
**Spermatogenic patterns of H4K5 acetylation and butyrylation during normal spermatogenesis.** (**A**) H4K5ac and H4K5bu H-scores represented as mean value ± SD per testicular cell type. *: *p*  <  0.05, **: *p*  <  0.01, ***: *p*  <  0.001, ****: *p*  <  0.0001. Results for H4K5ac represented in dark grey and for H4K5bu in green. (**B**) IHC images of H4K5ac and H4K5bu detection (brown-green color) in patients with normal spermatogenesis. Tissues were counterstained with PAS-haematoxylin. Scale bars = 10 µm. SPG: spermatogonia; SPC: spermatocytes; rSPD I–V: early-stage (I–V) round spermatids; rSPD VI–VIII: late-stage (VI–VIII) round spermatids; eSPD: elongating spermatids; cSPD: condensed spermatids; SC: Sertoli cells.

After strict anatomopathological evaluation of the testicular biopsies, testicular sections from patients displaying normal spermatogenesis (control group, N) were subjected to immunohistochemistry (IHC) analysis to detect H4K5 butyrylation and acetylation (Table 1 and Figure 1). The H4K5ac H-Score (expressed as mean value ± SD), which combines the number of positive cells and signal intensity, evidenced mild levels in spermatogonia (SPG, H-Score = 245.3 ± 59.8) that significantly decreased in spermatocytes (SPC, H-Score = 46.8 ± 29.1, *p* < 0.001) to finally recover intermediate levels as spermiogenesis started. Specifically, early-stage (I to V) round spermatids (rSPD I–V) restored the H-Score to 282.7 ± 66.2 (*p* < 0.0001), which increased to 415.8 ± 80.8 (*p* < 0.05) in late-stage rSPD (rSPD VI–VIII) maintaining similar levels in elongated spermatids (eSPD). H4K5ac levels finally decreased to the minimum in condensed spermatids (cSPD, H-Score = 0.9 ± 2.1, *p* < 0.0001). Somatic Sertoli-cells (SC) displayed low levels of H4K5ac (Table 1 and Figure 1). In turn, H4K5bu evidenced minimum H-Scores during initial stages of spermatogenesis up to rSPD VI–VIII (33 ± 35.4 in SPG, 0.0 ± 0.0 in SPC, and 30.8 ± 46.9 in rSPD I–V). A striking rise of the H4K5bu H-Score was observed in rSPD VI–VIII (319.1 ± 128.0, *p* < 0.01), reaching maximum levels in eSPD (as of stage IX spermatids, 478.4 ± 40.7, *p* < 0.05). Finally, H4K5bu levels decreased in cSPD (0.9 ± 2.1, *p* < 0.0001). SC showed an H4K5bu H-Score close to zero (2.5 ± 6.1) (Table 1 and Figure 1). Individual H4K5ac and H4K5bu H-Scores per patient are shown in Tables S1 and S2, respectively. Representative IHC images in testicular sections corresponding to patients with normal spermatogenesis are shown in Figure 1B (H4K5bu) and 1C (H4K5ac).

### 2.2. H4K5 Butyrylation Spermatogenic Levels Are Not Impaired under Different Testicular Defects

**Figure 2:**
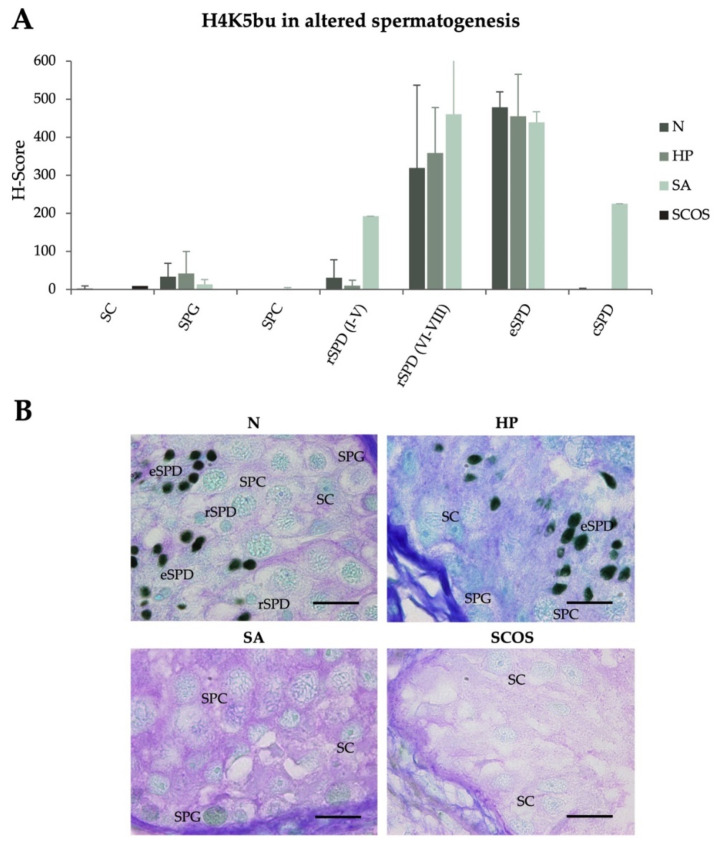
**Detection of H4K5 butyrylation levels in testicular biopsies of patients with altered spermatogenesis**. (**A**) H4K5bu H-scores of the normal spermatogenesis group (N), hypospermatogenesis (HP), spermatogenic arrest (SA), and Sertoli-cell only syndrome (SCOS), represented as mean value ± SD per testicular cell type. (**B**) Microscopy images showing H4K5bu detection in testicular section of patients affected with different spermatogenic alterations, compared to normal spermatogenesis. Tissues were counterstained with PAS-haematoxylin. Scale bars = 10 µm. SPG: spermatogonia; SPC: spermatocytes; rSPD: round spermatids; eSPD: elongating spermatids; SC: Sertoli cells.

After observing the predominant detection of H4K5bu in the late stages of spermatogenesis, we explored the characteristic behavior of this modification in patients displaying different primary testicular failures, such as hypospermatogenesis (HP), spermatogenic arrest (SA), and Sertoli-cell only syndrome (SCOS), aiming to identify potential atypical H4K5bu signals in meiotic or mitotic phases, or in supporting somatic SC. H4K5bu H-Scores were analyzed and are shown in Figure 2 and gathered in Table 1 (see Table S2 for detailed information per individual patient). No significant changes in the normal H4K5bu spermatogenic pattern were detected associated with spermatogenic alterations. Notably, a decrease in H4K5bu in rSPD I–V of patients with hypospermatogenesis and an increase in rSPD I–V and cSPD in patients with SA seems to occur. However, due to the limited number of samples in these two groups, we could not perform statistical analyses. Interestingly, the altered levels observed in the SA group are due to the contribution of a single patient with partial rSPD arrest (Table S2).

### 2.3. Testicular Cancers Do Not Affect the Spermatogenic Pattern of Butyrylation on H4K5, but Neither Acetylation on the Same Residue, in Healthy Seminiferous Tubules with Complete Spermatogenesis

**Figure 3:**
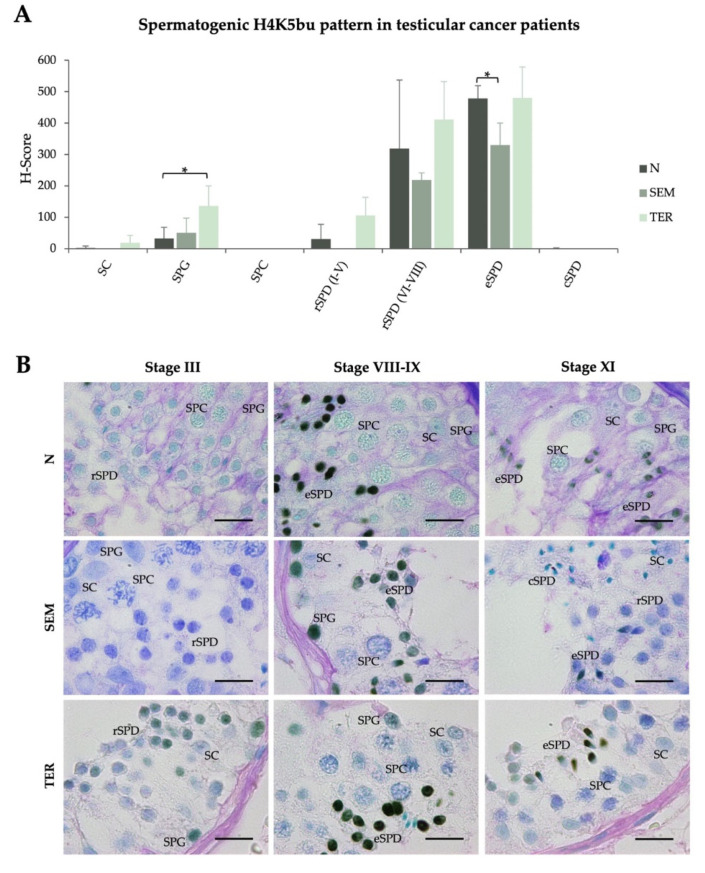
**Spermatogenic pattern of H4K5 butyrylation in seminiferous tubules of testicular cancer patients**. (**A**) H4K5bu H-scores (mean value ± SD) per testicular cell type corresponding to the normal spermatogenesis group (N), and patients with complete spermatogenesis but affected by seminoma (SEM) or teratoma (TER). (**B**) Microscopy images showing H4K5bu detection in testicular sections of SEM and TER patients, compared to healthy controls with normal spermatogenesis (N). Tissues were counterstained with PAS-hematoxylin. Scale bars = 10 µm. SPG: spermatogonia; SPC: spermatocytes; SPD: spermatids; SC: Sertoli cells. *: *p*  <  0.05.

H4K5bu spermatogenic pattern was also evaluated in patients affected by testicular cancers, such as seminoma (SEM) and teratoma (TER), to identify potential epigenetic alterations. TER patients showed a slight increase of the H4K5bu H-score in SPG (135.9 ± 64.3, *p* < 0.05) (Table 1, Figure 3A). In turn, SEM patients did not evidence significant changes in the H4K5bu spermatogenic pattern apart from a decrease in rSPD (330.1 ± 69.9, *p* < 0.05) (Table 1, Figure 3A). Despite these significant changes, the behavior of the H4K5bu spermatogenic pattern found in males with normal spermatogenesis, consisting in low levels at the beginning of the process, an increase from rSPD to eSPD, and a decrease in cSPD, is maintained in the testicular cancer patients included in this study (Figure 3A). Data corresponding to each individual patient and the IHC representative images are shown in Table S2 and Figure 3B, respectively.

**Figure 4:**
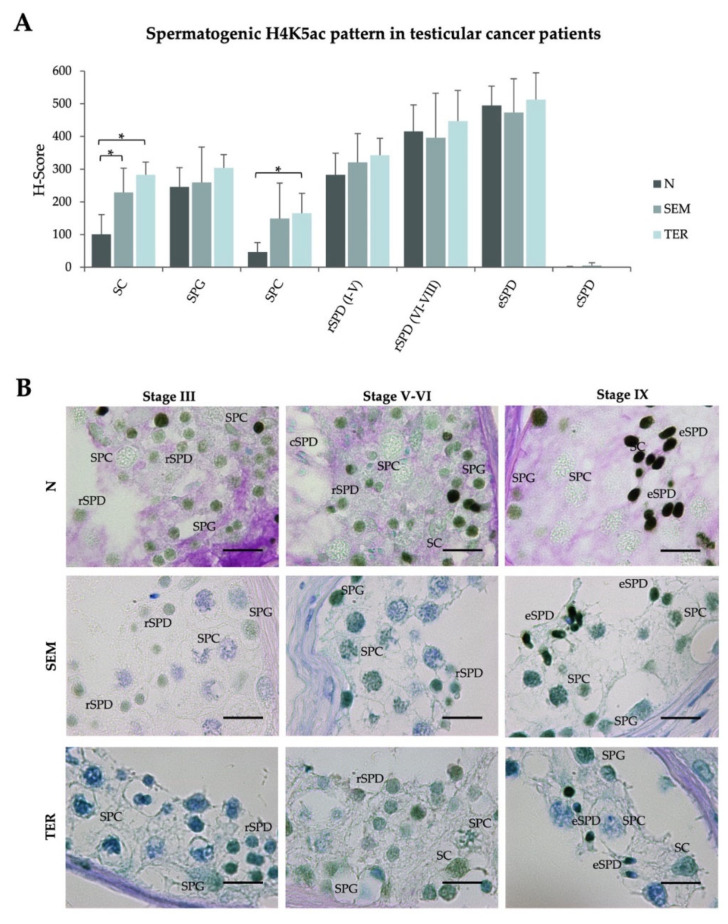
**Spermatogenic H4K5 acetylation levels in seminiferous tubules of testicular cancer patients**. (**A**) H4K5ac H-scores (mean value ± SD) per testicular cell type corresponding to the normal spermatogenesis group (N), and patients with complete spermatogenesis but affected by seminoma (SEM) or teratoma (TER). (**B**) IHC images corresponding to H4K5bu detection in testicular sections of SEM and TER patients, compared to healthy controls with normal spermatogenesis (N). Tissues were counterstained with PAS-hematoxylin. Scale bars = 10 µm. SPG: spermatogonia; SPC: spermatocytes; SPD: spermatids; SC: Sertoli cells. *: *p*  <  0.05.

Considering the relevance of global spermatogenic H4 acetylation in testicular cancer patients, which is completely disturbed in the seminiferous tubules adjacent to the tumor, we proceeded to evaluate specific acetylation of H4K5 in SEM and TER patients. Of note, significant changes to the normal pattern were only identified in SPC from TER patients, evidencing a discreet increase of H4K5ac H-score (165.5 ± 60.4, *p* < 0.05) (Table 1, Figure 4A). Despite that SEM patients displayed similar H4K5ac spermatocytic levels, the increase was not significant. Moreover, the H4K5ac normal spermatogenic pattern in which mild SPG levels decrease to SPC, to rise up to a maximum level in late round/early condensing spermatids followed by a final decrease in cSPD, is maintained in testicular sections of SEM and TER patients (Figure 4A). Somatic Sertoli cells showed higher H4K5ac levels in both groups (SEM: 228.5 ± 74.8, *p* < 0.05; TER: 282.5 ± 39.2, *p* < 0.05) Representative IHC images are shown in Figure 4B, and data corresponding to each individual patient are included in Table S2.

### 2.4. Butyrylation and Acetylation on H4K5 Are Retained in Ejaculated Human Sperm from Normozoospermic Patients

**Figure 5:**
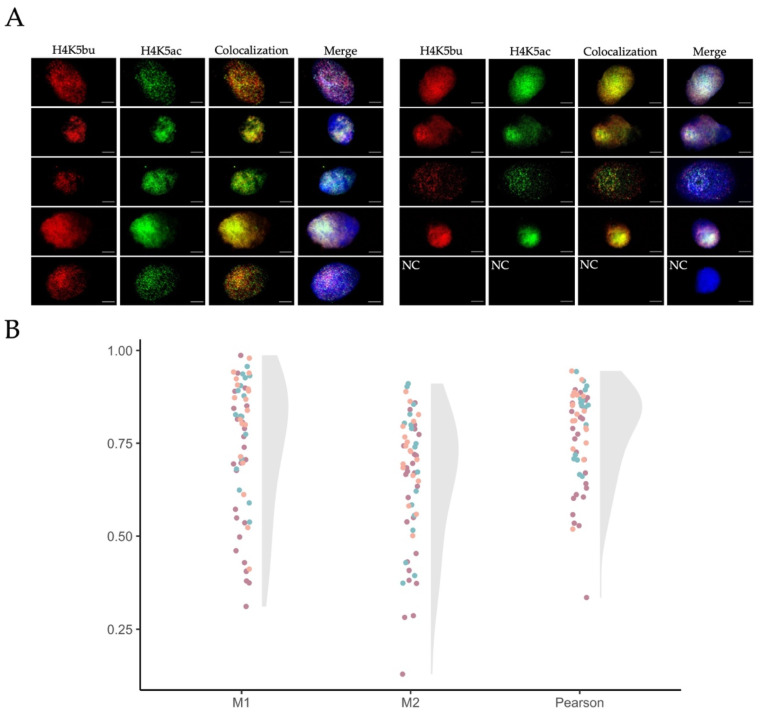
**Detection and colocalization of H4K5bu and H4K5ac in human mature sperm by immunofluorescence.** (**A**) Representative confocal microscopy images of H4K5bu (Alexa Fluor 633, red channel) and H4K5ac (Alexa Fluor 488, green channel) of the analyzed mature sperm nuclei under decondensed chromatin conditions. Scale bars = 5 µm. Sperm DNA is marked with DAPI (blue) and shown in Merge. NC indicates negative control. (**B**) Raincloud plot of the different colocalization coefficient parameters (“M1” and “M2” correspond to Mander’s colocalization coefficients M1 and M2, respectively, and “Pearson” to Pearson’s coefficient). Dots indicate individual sperm cells, and colors differentiates between biological replicates (*n* = 3).

To further investigate H4K5 butyrylation in male germ cells, we examined its presence and that of the acetylation on the same residue in mature human sperm from patients with normal semen parameters. Both H4K5bu and H4K5ac were detected in all ejaculated sperm cells evaluated from the patients included in the analysis (Figure 5A). To deepen into the interplay among the two PTMs, nucleosome clusters marked by either H4K5bu or H4K5ac were colocalized using confocal imaging (Figure 5B). Mander’s colocalization coefficient M1, representing detection of H4K5bu (red staining) over H4K5ac (green staining), corresponded to 0.74 ± 0.18, while M2 coefficient (H4K5ac over H4K5bu) was 0.65 ± 0.17 (mean value ± SD, Figure 4B). These results evidenced that around 70% of the nucleosome clusters marked by H4K5bu colocalized with those containing H4K5ac in the mature sperm head. The remaining 30% of the nucleosome clusters were exclusively marked by one of the epigenetic marks. Pearson’s coefficient was 0.78 ± 0.12, indicating a high direct correlation between the intensities of both PTMs.

### 2.5. The Proportion of H4K5bu over H4K5ac Remains Constant within Different Sperm Populations Selected According to Sperm Quality

**Figure 6:**
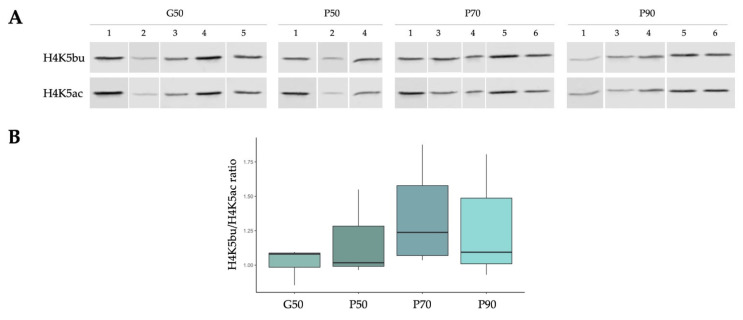
**H4K5bu/H4K5ac ratios based on signal intensity from fluorescent WB analysis.** (**A**) WB images. Sperm populations are indicated on top of the figure, and sample number is shown below to identify the different patients analyzed. Cell populations positive for leukocyte contamination (*PTPRC* cDNA, Ct < 35) or not fitting linearity threshold in both antibodies were discarded from the analysis. (**B**) Boxplot of the ratios according to the sperm population after density gradient separation.

A 50% density gradient centrifugation, which allows obtaining purified sperm representing the total sperm population of a semen sample without the presence of non-sperm contaminating cells (G50), was performed in order to define the relative abundance of both marks in normozoospermic samples (represented as H4K5bu/H4K5ac ratio). Moreover, a 3-layered (50–70–90%) density gradient centrifugation was performed to compare cell populations according to sperm quality (P50, P70, and P90, respectively). The presence of H4K5bu and H4K5ac was detected in all cell populations evaluated in our experimental set up. When considering the whole semen sample (G50), the relation H4K5bu/H4K5ac was established as 1.02 ± 0.1 (Figure 6). This ratio seems to increase in selected sperm populations, being 1.18 ± 0.32 in P50, 1.36 ± 0.36 in P70, and 1.27 ± 0.37 in P90. However, these differences were not significant among groups (*p* > 0.05), which might be due to the high dispersion of the results observed after the multi-layer gradient preparation, compared to the 50% gradients.

## 3. Discussion

Despite that H4 acetylation has been largely described during mammalian spermatogenesis and has shown to be directly involved in chromatin remodeling during spermiogenesis [9,10,13,29,31,32], similar description of other H4 acylations, such as butyrylation, is still scarce, especially in humans. The present study provides the first characterization of H4K5 butyrylation during human spermatogenesis and in mature sperm, in comparison with the functionally close-related acetylation of the same residue (H4K5ac). We report a different behavior among both PTMs suggesting a highly specific role of H4K5bu in spermatid elongation, temporarily coexisting with the process of histone to protamine transition, that is not altered in patients with testicular conditions. Strikingly, while global H4ac levels are fully disturbed in the presence of testicular cancer [32], neither H4K5bu nor H4K5ac were found altered in seminiferous tubules adjacent to the tumoral tissue in this study. Therefore, this residue may be somehow resistant against testicular perturbations, compatible with a proper NH-NP transition. Furthermore, both H4K5 PTMs are retained in normal human mature spermatozoa, which opens a window to study the potential of this residue as an epigenetic mark delivered into the zygote.

The spermatogenic pattern of H4K5ac found in this study was consistent to that of global H4ac previously described [29,30,31,32]. SPG displayed intermediate levels of H4K5ac, which decrease in SPC and are restored in rSPD I–V. During rSPD development, H4K5ac levels gradually increased to reach a maximum value in eSPD. Strikingly, the H4K5bu spermatogenic pattern showed a different behavior, with basal butyrylation levels during the first phases of spermatogenesis and initial stages of round spermatid. Additionally, the drastic increase in H4K5bu levels starts in a more advanced stage of rSPD than H4K5ac does, temporarily coexisting with spermatid elongation when NH-NP transition occurs. Thus, H4K5bu seems to show a highly specific role during the latest steps of human spermatogenesis, coherent with previous reports in mouse models suggesting a temporal regulation of nucleosome disassembly and histone retention based on histone butyrylation [36]. Despite the low immunohistochemical levels of H4K5bu in early spermatogenesis (null in SPC) reported herein, we do not discard that H4K5bu in SPC could be better characterized using more sensitive techniques, as previously reported through western blot and mass spectrometry in sorted mouse spermatogenic germ cells [36].

Due to the apparent highly specific role of H4K5bu during spermiogenesis, we explored whether butyrylation levels were altered under different primary testicular failures. No relevant changes were observed in patients with hypospermatogenesis, SCOS, or spermatogenic arrest, as previously reported for H4ac [32]. Despite that, a patient with partial rSPD arrest displayed increased H4K5bu levels in rSPD I–V and cSPD. This could suggest that even though H4K5bu is not altered nor has a role in meiotic arrest, its alteration could either be involved in the spermatid arrest or be its consequence, which would be coherent with the potential specific role of this PTM observed in normal spermiogenesis. The lack of statistical analysis due to sample number availability limits the interpretation of this finding, but the spermatogenic arrest in post meiotic stages seems to be an interesting phenotype to study H4K5 PTMs in subsequent studies. We were interested in exploring H4K5 PTMs in cancer patients since a drastic dysregulation of the H4ac levels has been recently reported in patients with different types of testicular tumors [32]. However, H4K5bu does not seems to undergo such drastic global increase in patients with testicular cancer, since only TER patients evidence a discrete increase of H4K5bu H-scores in SPG. Nevertheless, in the present work, only butyrylation in a single residue of H4 has been studied, which does not discard different results when analyzing global H4bu or lysine butyrylation, as observed for H4ac [32]. It is interesting to remark that spermatogenic H4K5ac levels were not found altered in cancer patients, except from a slight increase in SPC from TER patients. These results contrast with the globally altered H4ac levels in testicular cancer patients reported elsewhere [32], which might be due to altered acetylation on other H4 residues. The stable levels regardless of testicular alterations point out a highly conserved role of H4K5bu and H4K5ac towards a proper regulation of spermiogenesis and could explain why testicular cancer patients, although having global H4ac disruption, display complete spermatogenesis. These data represent the first joint characterization in human subjects of acetylation and butyrylation on H4K5 during spermatogenesis. Although the number of individuals analyzed could limit the interpretation of the significant differences reported herein, our results are consistent with previous studies in mouse spermatogenesis [36].

Growing evidence highlights the impact of sperm chromatin state in sperm functionality, fertilization events, and beyond, including sperm modified histones as epigenetic marks inducing differential DNA accessibilities [2,4,5,37,38,39,40,41,42,43,44,45]. Despite the observed drastic drop of the IHC H4K5bu and H4K5ac levels in cSPD, both were found retained in mature sperm from normozoospermic patients, colocalizing in around 70% of nucleosome clusters. This high colocalization percentage might be due to the rapid acetylation and butyrylation turnover taking place in H4K5 during spermatogenesis [36], resulting in such “photo-finish” in the transcriptionally and translationally silent sperm cell. Since two H4s are present in the histone octamer conferring the nucleosome, two different scenarios arise from this co-localization: (1) the presence of single nucleosomes containing one H4 marked by butyrylation on K5 and the other by acetylation on the same residue, or (2) nucleosomes with both histones H4 marked by either butyrylation or acetylation on K5, that are spatially close to nucleosomes with the alternative mark, making the signals coexist. Since single nucleosomes are not resolved at this confocal imaging resolution, both possibilities are equally feasible, again supported by the rapid H4K5ac and H4K5bu turnover in the testis. Interestingly, the remaining proportion of non-colocalizing pixels detected in this study also suggest the presence of a group of nucleosome clusters exclusively marked by either H4K5bu or H4K5ac. This data might point to a functional involvement of the differential wrapping of paternal DNA into H4K5ac or H4K5bu nucleosomes.

While colocalization results might suggest a role of H4K5ac and H4K5bu in sperm function, no variations were observed associated with sperm quality. The study of different sperm populations isolated from neat sample following procedures routinely used in the reproductive clinics to select the best spermatozoon for ICSI sheds light on the molecular cargo of the fertile sperm giving rise to a new individual. In that case, the observed results would discard an impact of H4K5bu and H4K5ac relative levels in sperm fertilizing ability. Further work should focus on determining quantitative H4K5bu and H4K5ac levels in selected sperm populations by complementary techniques, which would help disentangle their individual potential as an indicator of chromatin maturity. Due to the known role of sperm histone marks as epigenetic regulators in early embryo, it will be worthy to know whether they could have an impact beyond fertilization, such as in the formation of preimplantation embryo and transmission of epigenetic information.

## 4. Materials and Methods

### 4.1. Biological Material

#### 4.1.1. Testicular Biopsies

Testicular biopsies (*n* = 23, mean age 37 ± 7, ranging from 21 to 52 years old) were provided by the Departments of Pathology from the Hospital Clínic de Barcelona (Barcelona, Spain) and the Hospital Universitari de Vic (Vic, Spain). The samples were grouped into three main groups: control group, spermatogenic alterations, and testicular cancer. The control group (N) comprised azoospermic infertile patients corresponding to male partners of couples undergoing assisted reproduction studies, displaying normal spermatogenesis at the histological level, and showing all germ cell types and spermatogenic stages. The origin of the azoospermia was classified as post-vasectomy (*n* = 1), obstructive azoospermia due to CFTR genotypes (*n* = 1), and azoospermia of unknown cause when obstructive azoospermia, karyotype alterations, CTCF pathogenic variants, and chromosome Y microdeletions were discarded (*n* = 4). H4K5ac and H4K5bu levels were assessed in 6 N samples, from which 5 were common to both evaluations, and 1 patient was exclusively subjected to either H4K5bu or H4K5ac assessment. The group of infertile patients with spermatogenic alterations (*n* = 8) included patients stratified according to the type of spermatogenic impairment into patients with Sertoli-cell only syndrome (SCOS; *n* = 3), hypospermatogenesis (HP; *n* = 2), and spermatogenic arrest (SA; *n* = 3). Among the SA patients, two of them presented spermatocyte arrest (one complete arrest and other partial arrest), and the third one evidenced a partial arrest at round spermatid level. The last group of patients with testicular cancer (*n* = 7) included patients affected by seminoma (SEM; *n* = 4) and teratoma (TER; *n* = 3). In addition to the groups above, one sample corresponding to a patient with normal spermatogenesis was used as technical internal control in all evaluations. Karyotype, AZF status, and CFTR status per individual patient are indicated in Tables S1 and S2.

#### 4.1.2. Semen samples

Human normozoospermic semen samples (*n* = 9) from patients undergoing routine semen analysis were obtained at the Assisted Reproduction Unit from the Clinic Institute of Gynecology, Obstetrics and Neonatology, at the Hospital Clínic de Barcelona, Spain. The ejaculates were collected by masturbation into sterile containers after 3–5 days of sexual abstinence. Evaluation of the seminal parameters was performed using the automatic semen analysis system CASA (Proiser, Paterna, Spain) and samples were classified according to the World Health Organization guidelines [51]. Sperm cells were purified through density gradient centrifugation, depending on the purpose of the analysis. A 50% Puresperm density gradient separation (NidaCon International AB, Gothenburg, Sweden), following manufacturer’s instructions, was used to analyze purified sperm representing the heterogeneity of the native sample and avoiding contamination by cells other than sperm. Three-layered 50–70–90% Puresperm density gradient separation (NidaCon International AB), was used to separate different sperm populations according to sperm quality for subsequent comparative studies. To discard the presence of somatic contamination that could alter the results, RNA expression analysis of the leukocyte marker receptor-type tyrosin-protein phosphatase C (PTPRC) through RT-PCR was performed to verify the absence of leukocytes according to Jodar et al., 2012 [52] (forward primer: CCTTGAACCCGAACATGAGT, reverse primer: ATCTTTGAGGGGGATTCCAG, corresponding to exons 12–13 of *PTPRC*).

### 4.2. Processing and Paraffin-Embedding of the Testicular Material

According to the hospital’s routine procedures, samples were fixed prior to paraffin-embedding in either Bouin’s fixative for 3–4 h or 4% Formol for 24–48 h, depending on sample size (biopsies or orchiectomy specimens, respectively), for histopathologic evaluation or other procedures. Samples from infertile patients, either from the control or spermatogenic alteration groups, were biopsied. Orchiectomy specimens were obtained from testicular cancer patients. After fixation, the specimens were processed in a fluid-transfer advanced automatic tissue processor and paraffin-embedded to make the blocks. The tissue was covered in molten paraffin in a mold and paraffin cooled down until solidifying. The block was cut through microtome into 4 µm-sections.

### 4.3. Testicular Histopathological Evaluation

An experienced anatomopathologist exhaustively performed the histopathological evaluation of the hematoxylin-eosin-stained testicular sections from each patient to identify spermatogenic defects [53] and/or testicular tumors, following the World Health Organization (WHO) guidelines [54,55]. Tumor diagnosis was confirmed in case of need by checking expression of appropriate biomarkers. Ultrasound evaluation was monitored to discard the presence of testicular microlithiasis [56]. In the group of testicular cancer patients, the presence of either complete or partial spermatogenic activity in seminiferous tubules adjacent to the neoplastic area was a strict inclusion criterion. After histopathological confirmation, additional testicular sections from each patient were used for further immunohistochemical analyses.

### 4.4. Immunohistochemistry

Immunohistochemistry (IHC) was performed on paraffin-embedded testicular sections, as described previously [32]. Testicular tissue samples were randomly grouped in different runs, and an internal positive control was included in each of them to check the different runs’ overall intensity. Due to the use of different fixation methods, effect of the fixative was discarded by checking that no changes in the spermatogenic pattern with the antibodies of interest were detected between an orchiectomy specimen fixed with 4% Formol and a biopsy fixed with Bouin, both showing normal spermatogenesis and no presence of testicular tumor (Figure S1).

Sections were dewaxed in toluene and rehydrated through graded series of ethanol to water with 0.3% hydrogen peroxide incubation. Subsequently, antigen retrieval was performed with 10 mM sodium citrate (pH 6.0) at 99.5 °C for 20 min. Slides were then blocked with PBS-5% skim milk for 30 min at room temperature (RT) and incubated with the Avidin/Biotin Blocking Kit (Vector Laboratories, Burlingame, CA, USA). Sections were incubated overnight at 4 °C with primary antibodies (dil 1:100, Table 2). Negative controls without primary antibody were included. Sections were incubated with biotinylated secondary antibody (Table 2) and an avidin-biotin-peroxidase detection kit (Vectastain ABC Elite Kit, Vector Laboratories). Signal color was developed by reaction with diaminobenzidine (DAB, 3,3′-diaminobenzidine tetrahydrochloride) and sections were counterstained with hematoxylin and Periodic Acid-Schiff’s (PAS) reagent (Sigma-Aldrich, St. Louis, MO, USA). Sections were dehydrated in ethanol, cleared in toluene, and mounted in Eukitt Mounting Medium (Sigma-Aldrich) to be analyzed in a transmission light microscope (Olympus BX50, Olympus, Tokyo, Japan).

### 4.5. Spermatogenic Stage Classification and IHC Evaluation

Classification of testicular cells and spermatogenic stages of the seminiferous tubules sections was based on current classification of human spermatogenesis described by Muciaccia et al. 2013 [57]. For each patient, at least 30 seminiferous tubule sections at different spermatogenic stages were analyzed. IHC was assessed by two independent operators using PAS–Haematoxylin staining, blinded to the experimental procedures. H-score was selected as the scoring system [32]. Specifically, the H-score was determined by adding the proportion of cells presenting each level of intensity (expressed in percentage) multiplied by the intensity (expressed in ordinal values) as follows: H-score = (%_intensity 0_ × 0) + (%_intensity 1_ × 1) + (%_intensity 2_ × 2) + […], presenting a dynamic range from 0 to 600. Since the intensity of the signal within cells belonging to the same specific cell type per spermatogenic stage was homogeneous along the seminiferous tubule section, the mean intensity of each cell type was considered. Thus, H-score for each testicular cell type analyzed per seminiferous tubule section was provided for each individual patient.

### 4.6. Immunofluorescence on Mature Sperm Cells

Extensions consisting in 1 million sperm (Mz) per slide were incubated in decondensing solution (2,5 mM DTT, 0,2% Triton X-100, 100 UI/mL heparine in PBS) to facilitate antibody accessibility, monitoring decondensation in optic microscope. Sperm cells were fixed 10 min in 2% paraformaldehyde and washed with PBS and H_2_O before permeabilization for 20 min in 0.05% Triton X-100 in PBS. After washings in PBS, slides were incubated for 1h at RT in blocking solution (5% bovine serum albumin in PBS). Primary antibody incubation (Table 2, dil 1:100) was performed overnight (O/N) at 4 °C. After washes, slides were incubated with secondary antibody (Table 2) at dil 1:500 for 2h at RT. After washings, slides were incubated with DAPI 1:10,000 for 5 min protected from light and mounted with ProLong^TM^ Gold (#P36930, Invitrogen, Waltham, MA, USA). Images were acquired by confocal microscopy (LSM880, ZEISS, Jena, Germany) at the Advanced Optical Microscopy Unit (Campus Clínic) from Scientific and Technological Centers from University of Barcelona.

### 4.7. Colocalization Analyses

Colocalization was quantified using ImageJ software distribution FIJI [58]. A custom macro was developed to automatize colocalization analysis with JACOP [59] for every set of images. Briefly, Red and Green Channel Images were background-subtracted with Rolling Ball Radius of 50 and colocalization was quantified from a region of interest delimiting the cell contour, considering a colocalized point if its respective intensities are strictly higher than the threshold determined for its channels (auto threshold method). Pearson correlation and Manders coefficients were analyzed from each image using JACOP plugin.

### 4.8. Odyssey^®^ Western Blot

Sperm soluble protein extraction was conducted by incubating purified sperm cells in lysis buffer (2% SDS, 1 mM PMSF in PBS, ratio 40 Mz:100 µL) for 15 min on ice followed by centrifugation 17,000× *g* for 10 min at 4 °C. Quantification was done using Pierce™ BCA Protein Assay Kit (Thermo Fisher Scientific, Rockford, IL, USA), following the manufacturers’ recommendation. Replicates with Ct < 35 for the leukocyte marker PTPRC were discarded. A total of 10 µg of soluble protein lysate were loaded into 16% acrylamide gels by SDS-PAGE [5]. Proteins were transferred to PVDF 0.2 µm pore size membranes (BioRad, Hercules, CA, USA), activated with methanol, in transference buffer (200 mM glycine, 25 mM Tris, 15% methanol). Membranes were shortly washed with 100% ethanol prior blocking with Intercept^®^ (TBS) blocking buffer (LI-COR Inc., Lincoln, Nebraska) for 1 h at RT to incubate O/N with primary antibodies at 4 °C (1:1000, Table 2). After washes with TBT (0.242% Tris, 0.8% NaCl, 0.1% Tween-20), membranes were incubated with secondary antibodies (Table 2) at 1:12,000 dilution for 1h at RT. Subsequent washes with TBT and TBS (20 mM Tris, 150 mM NaCl, pH 7.6) and fixation with pure methanol were conducted and the membrane was air-dried. Imaging was performed using Odyssey^®^ infrared analysis system, with laser intensities 700 = 2.0, 800 = 3.0, according to the best fit for antibodies’ linearity (R^2^ > 0.98). Signal intensities were determined on the raw scanned data using Image Studio Lite v5.2 software (LI-COR Inc.). Intensities below the minimum linear cutoff were discarded from the analysis. Therefore, after exclusion criteria, from the 6 patients analyzed, *n* = 5 in G50, P70, and P90 and *n* = 3 in P70 were considered for subsequent statistical analysis.

### 4.9. Statistical Analysis

Data analysis was conducted in RStudio software version 2022.02.2 [60] and the car package [61], and using GraphPad Prism version 7.0 for Windows (GraphPad Software, La Jolla, CA, USA, www.graphpad.com). Normal distribution was assessed by Shapiro–Wilk normality test followed by Levene’s homoscedasticity test. According to the distribution, either Student t-test or Mann–Whitney U test was performed for the analysis of H-Score levels. Only groups formed by at least three patients were included in the statistical analysis. Paired Student t-test was applied for the analysis of H4K5bu/ac ratios. *p*-values < 0.05 were considered statistically significant.

## Figures and Tables

**Table 1:** **IHC H-Scores detected in the different cells from the seminiferous tubules according to the group of patients.** H-Scores (mean ± SD) correspond to H4K5ac and H4K5bu in control and testicular cancer patients and H4K5bu in patients with altered spermatogenesis.

		H-Scores (Mean ± SD)						
		SC	SPG	SPC	rSPD (I–V)	rSPD (VI–VIII)	eSPD	cSPD
H4K5bu								
Infertile patients with normal spermatogenesis	**N (*n* = 6)**	**2.5 ± 6.1**	**33 ± 35.4** **^#^**	**0.0 ± 0.0**	**30.8 ± 46.9**	**319.1 ± 128.0** **^#^**	**478.4 ± 40.7** **^#^**	**0.9 ± 2.1** **^#^**
Infertile patients with altered spermatogenesis	**SCOS (*n* = 3)**	8.9 ± 15.4	-	-	-	-	-	-
	**HP (*n* = 2)**	0.0 ± 0.0	41.3 ± 58.4	0.0 ± 0.0	10.0 ± 14.14	358.9 ± 118.7	455.6 ± 110.0	0.0 ± 0.0
	**SA (*n* = 3)**	0.0 ± 0.0	12.8 ± 13.2	1.7 ± 3.3	192.3	460.0 ± 242.5	439.4 ± 27.4	225.0
Testicular cancer patients	**SEM (*n* = 4)**	0.0 ± 0.0	51.2 ± 46.3	0.0 ± 0.0	0.0 ± 0.0	219.0 ± 22.9	330.1 ± 69.9 *	0.0 ± 0.0
	**TER (*n* = 3)**	18.7 ± 23.8	135.9 ± 64.3 *	0.0 ± 0.0	106.2 ± 57.3	411.1 ± 120.6	480.2 ± 98.7	0.0 ± 0.0
**H4K5ac**								
Infertile patients with normal spermatogenesis	**N (*n* = 6)**	**101.1 ± 59.5**	**245.3 ± 59.8**	**46.8 ± 29.1** **^#^**	**282.7 ± 66.2** **^#^**	**415.8 ± 80.8** **^#^**	**494.9 ± 59.4**	**0.9 ± 2.1** **^#^**
Testicular cancer patients	**SEM (*n* = 4)**	228.5 ± 74.8 *	259.2 ± 108.2	149.4 ± 107.9	320.7 ± 88.4	396.5 ± 135.9	473.4 ± 102.8	5.7 ± 7.86
	**TER (*n* = 3)**	282.5 ± 39.2 *	304.3 ± 40.2	165.5 ± 60.4 *	342.9 ± 51.5	447.2 ± 93.7	513.1 ± 81.7	0.0 ± 0.0

^#^: *p* < 0.05 after comparison of H-Scores between consecutive spermatogenic cell types in normal patients (N). *: *p*  < 0.05 after comparison of each testicular cell type between H-Scores of patients with testicular pathologies and the control group. For H4K5bu comparisons, Student t-test was performed in rSPD (VI–VIII) and eSPD, while Mann–Whitney U test was applied in the rest of the cell types. H4K5ac comparisons were performed through Student t-test in all cell types except SPC and cSPD, in which Mann–Whitney U test was applied. Note that HP was not considered for statistical analysis due to the limited sample availability. The same applies to SA from the spermatogenic stage of rSPD (I–V), due to low number of replicates. Abbreviations: cSPD, condensed spermatids; eSPD, elongating spermatids; rSPD I–V, early-stages (I–V) round spermatids; rSPD VI–VIII, late-stages (VI–VIII) round spermatids; SC, Sertoli cells; SPC, spermatocytes; SPG, spermatogonia; N, normal spermatogenesis; SCOS, Sertoli-cell only syndrome; HP, hypospermatogenesis; SA, spermatogenic arrest; SEM, seminoma; TER, teratoma.

**Table 2:** **Antibodies used in the study**.

Antibodies	Company	Reference
**Primary antibodies**		
Rabbit monoclonal anti-H4K5bu	PTM BIO LLC, Chicago, IL, USA	#PTM-313
Mouse monoclonal anti-H4K5ac	PTM BIO LLC, Chicago, IL, USA	#PTM-163
**Secondary antibodies (IHC)**		
Goat Anti-Rabbit IgG Antibody (H + L), Biotinylated	Vector Laboratories, Burlingame, CA, USA	BA-1000
Goat Anti-Mouse IgG Antibody (H + L), Biotinylated	Vector Laboratories, Burlingame, CA, USA	BA-9200
**Secondary antibodies (IF)**		
Goat anti-mouse IgG Alexa Fluor 488	Invitrogen, Waltham, MA, USA	A28175
Goat anti-rabbit IgG Alexa Fluor 633	Invitrogen, Waltham, MA, USA	A21070
**Secondary antibodies (Odyssey^®^ Fluorescent WB)**		
Anti-Mouse IgG (IRDye-700)	LI-COR, Inc., Lincoln, NE, USA	P/N 925-68070
Anti-Rabbit IgG (IRDye-800)	LI-COR, Inc., Lincoln, NE, USA	P/N 925-32211

## Data Availability

Not applicable.

## Supplementary Materials

The following supporting information can be downloaded at: https://www.mdpi.com/article/10.3390/ijms232012398/s1.
