# Supplementary material for: H4K5 Butyrylation Coexist with Acetylation during Human Spermiogenesis and Are Retained in the Mature Sperm Chromatin
Source: Int J Mol Sci. 2022 Oct 17;23(20):12398. doi: 10.3390/ijms232012398 (PMC9604518; doi:10.3390/ijms232012398)
Supplement: Supplementary file 1 [file ijms-23-12398-s001.zip › Figure S1_ v20220831.pdf]

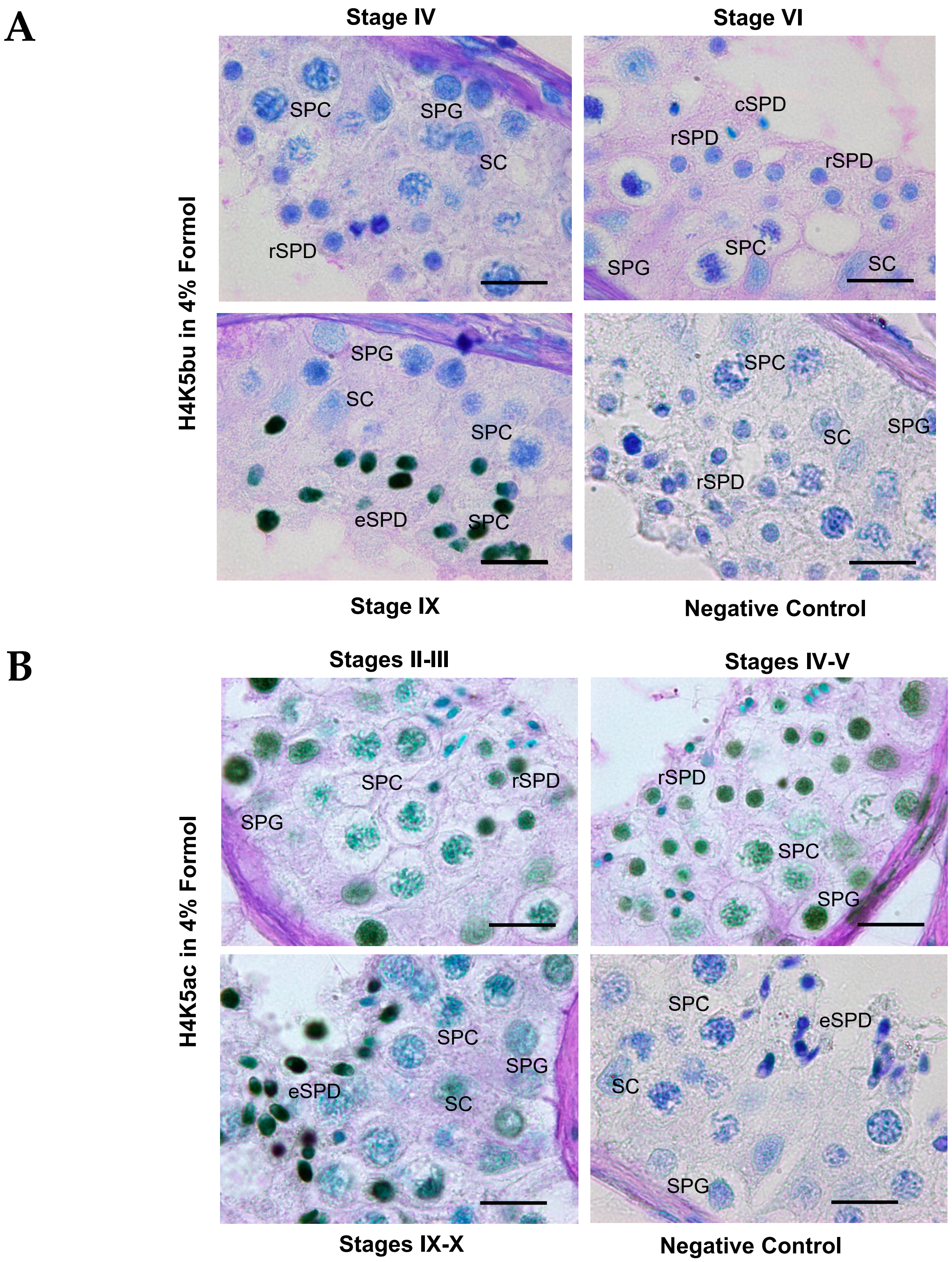

Figure S1. Spermatogenic patterns of H4K5 acetylation and butyrylation during normal spermatogenesis in an orchiectomy specimen fixed in 4% Formol. (**A**) and (**B**) show representative images of the IHC detection (brown color) of H4K5bu and H4K5ac, respectively, in patients with normal spermatogenesis. Results are comparable to the ones obtained in biopsies fixed with Bouin's fixative (Figure 1B). Tissues were counterstained with PAS-haematoxylin. Scale bars = 10 μm. SPG: spermatogonia; SPC: spermatocytes; rSPD I–V: early-stages (I–V) round spermatids; rSPD VI–VIII: late-stages (VI–VIII) round spermatids; eSPD: elongating spermatids; cSPD: condensed spermatids; SC: Sertoli cells.
